# Supplementary material for: Upregulation of PODXL and ITGB1 in pancreatic cancer tissues preoperatively obtained by EUS-FNAB correlates with unfavorable prognosis of postoperative pancreatic cancer patients
Source: PLoS One. 2022 Mar 11;17(3):e0265172. doi: 10.1371/journal.pone.0265172 (PMC8916642; doi:10.1371/journal.pone.0265172)
Supplement: Attachment — Submitted filename: (revise2)TANIUCHI et al_ResponseToReviewer.pdf [file pone.0265172.s002.pdf]

Dr. Surinder Batra,

December 14, 2021

Submission ID: PONE-D-21-14241R1

Upregulation of PODXL and ITGB1 in pancreatic cancer tissues preoperatively obtained by EUS-FNAB correlates with unfavorable prognosis of postoperative pancreatic cancer patients

Dear Dr. Surinder Batra,

I am returning the above manuscript that was revised according to your letter dated the 10th of November. Our responses to the reviewers' comments are provided below.

Additionally, the funders of "Grants-in-Aid for Scientific Research (KAKENHI): 17K09463 (kt), 19K07461 (mf), 20K07806 (ry), 20K08359 (kt) and 20K07699 (mo), and AMED under Grant Number JP19lm0203007 (kt)" had no role in study design, data collection and analysis, decision to publish, or preparation of the manuscript.

**Reviewer #2:**

We appreciate the Reviewer's useful comments.

Comments:

1. However, the use of TCGA data, while a meaningful addition, is not executed appropriately in this manuscript. In the TCGA PAAD dataset, there a number of benign and malignant entities which comprise the the 183 samples in the full data set. To indicate that these are all PDAC samples is misleading and incorrect. Moreover, the inclusion of pancreatic neuroendocrine tumors (included in the 183 samples) in the analysis has the potential to bias or confound the meaning of outcomes studies. Please repeat this analysis using only the samples in the TCGA PAAD dataset that are confirmed to be PDAC.

**Response**

In response to the Reviewer's comments, we excluded neuroendocrine tumors and repeated the prognostic analysis of 42 PDACs that were confirmed to be TNM stage IA-IIA using the TCGA bioinformatics platform. Based on Kaplan-Meier curves, the postoperative overall survival rate for PDAC patients with UICC TNM stage IA-IIA with upregulated mRNA levels of both PODXL and ITGB1 (n = 7) was significantly lower than that for PDAC patients without high expression of both PODXL and ITGB1 (n = 35) (P=0.006), as shown in Fig. 6B.

I hope that the revised manuscript is now acceptable for publication.

Yours sincerely,

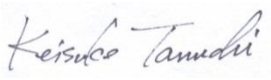

Keisuke Taniuchi, MD, PhD.
Department of Gastroenterology and Hepatology
Kochi Medical School, Kochi University
Kohasu, Oko-cho, Nankoku, Kochi 783-8505, Japan
Phone: +81-88-880- 2338; Fax: +81-88-880- 2338
Email: ktaniuchi@kochi-u.ac.jp
